# Prevalence of chronic complications of type 2 diabetes mellitus in outpatients - a cross-sectional hospital based survey in urban China

**DOI:** 10.1186/1477-7525-8-62

**Published:** 2010-06-26

**Authors:** Zhaolan Liu, Chaowei Fu, Weibing Wang, Biao Xu

**Affiliations:** 1 Department of Epidemiology, School of Public Health, Fudan University, Shanghai 200032, PR China; 2 Centre for Evidence-based Medicine, Beijing University of Chinese Medicine, Beijing 100029, PR China

## Abstract

**Background:**

Chronic complications are the major outcome of type 2 diabetes mellitus progress, which reduce the quality of life of patients, incur heavy burdens to the health care system, and increase diabetic mortality. The aims of this study were to describe the prevalence of chronic complications among urban Chinese type 2 diabetic outpatients; and to analyze the associations between chronic complications and patients' demographics, diabetic related clinical characteristics.

**Methods:**

This cross-sectional hospital-based study was carried out in 4 major Chinese cities: - Shanghai, Chengdu, Beijing and Guangzhou. The survey was conducted from March to July in 2007 among 1,524 type 2 diabetic outpatients. The subjects were interviewed face-to-face by trained interviewers using a questionnaire to capture information on demographics, disease presentations and complications. All the subjects were invited to have a HbA1c test free of charge by the standardized method with Bio-Rad Variant II.

**Results:**

Of the 1,524 study subjects, 637 (41.8%) were male, and the mean age was 63.3 ± 10.2 years. At least one chronic complication was diagnosed in 792 individuals (52.0%) of the study subjects; 509 (33.4%) presented with macrovascular complications and 528 (34.7%) with microvascular complications. The prevalence of cardiovascular and cerebrovascular conditions, neuropathy, nephropathy, ocular lesions and foot disease were 30.1%, 6.8%, 17.8%, 10.7%, 14.8% and 0.8%, respectively. The prevalence of chronic complications varied between cities, and significantly increased with age and duration of diagnosed diabetes. The mean of HbA1c in diabetic patients with chronic complications was 8.2% ± 1.6% and 63.0% of the subjects with type 2 diabetes related complications had a poor glycemic control with the HbA1c > 7.5%.

**Conclusions:**

Chronic complications are highly prevalent among type 2 diabetic outpatients, the glycemic control of diabetic patients with chronic complications was poor, and future efforts should be directed at intensive blood glucose control, strengthening early diagnosis and improving case management to prevent and minimize the occurrence of complications.

## Introduction

Globally, type 2 diabetes mellitus (T2DM) has become one of the most important chronic public health problems[1]. T2DM is a growing cause of disability and premature death, mainly through cardiovascular disease and other chronic complications[1-3]. It is estimated that the global number of adults suffering from any form of diabetes will reach 285 million in 2010 and further increase to 439 million in 2030, most of them T2DM cases[4,5]. China had an estimated 23.8 million diabetics in 2003, more than 92 million adults with diabetes as it was reported in 2010, and is considered one of the countries with the largest T2DM burden[1,6].

Data from prospective and cross-sectional studies consistently point to the fact that diabetic patients are more likely to develop micro- as well as macro-vascular conditions[7-9]. Prior to the onset of diabetes, many patients already show metabolic abnormalities, such as dyslipidemia, further contributing to the development of complications[8]. About 50% of the subjects of UKPDS had substantial macro- or micro-vascular abnormalities at the time of T2DM diagnosis[9]. It is well known that chronic complications are the major outcome of T2DM progress, which reduce the quality of life of patients, incur heavy burdens to the health care system, and increase diabetic mortality [10-12]. After adjusting for age, the death rate of people with T2DM is about twice as high as their non-diabetic peers[13]. About 50-80% of all individuals with diabetes die of cardiovascular disease, with cerebrovascular disease, and kidney failure also among the leading causes of death[1,13]. Permanent disability is a common outcome of diabetes, with late complications of diabetes being major determinants for disability. Diabetic eye disease, particularly retinopathy, has become a major cause of blindness throughout the world[1,14]. Moreover, clinical epidemiologic studies suggest that foot ulcers precede more than 85% of non-traumatic lower extremity amputations (LEAs) in diabetic individuals[15].

Access to diabetic care is limited in low and middle income countries (including China) where more than 70% of diabetic patients live [16]. As a transitional society, China is facing a rapid rise of the T2DM population accompanying its remarkable economic development, especially in urban populations. Yet, few studies have addressed the extent of the T2DM epidemic, as well as the disease burden of diabetic complications to China's health care system. It is obvious that information on prevalence of T2DM related complications is important for the adjustment of policies and practices in diabetic care management to gain better control of T2DM. The aims of this study were to describe the prevalence of chronic complications among urban Chinese T2DM outpatients; and to analyze the associations between chronic complications and patients' demographics, T2DM related characteristics.

## Methods

### Study population, recruitment and data collection

This cross-sectional study was carried out in 4 major Chinese cities representing the east, west, north and south of mainland China: Shanghai, Chengdu, Beijing and Guangzhou, respectively. Fifteen general hospitals with endocrinology departments offering specialized diabetes care were selected purposively according to the service coverage, capacity in treating diabetes and participation intention of the hospitals, with three to six in each city. The outpatients were consecutively recruited in each hospital. All T2DM outpatients fulfilling the inclusion criteria detailed below and attending the study facilities during the period March - July 2007 were invited to participate in the study.

The inclusion criteria were: (1) T2DM diagnosed in accordance with international standards (WHO 1999), i.e. fasting plasma glucose (FPG) ≥ 7.0 mmol/L and/or 2 hours postprandial plasma glucose (PPG) or casual plasma glucose ≥ 11.1 mmol/L[17]; (2) under regular anti-diabetic drug treatment for at least 1 year; (3) ≥ 18 years old; (4) resident in the respective city for ≥ 2 years; (5) provided written informed consent to participate in the study.

Detailed information regarding the study procedures was provided to all eligible individuals. Only patients who agreed to participate and signed the consent form were included in the study. For those refused to participation, no further information was collected. The subjects were interviewed face-to-face by trained interviewers using a questionnaire to capture information on demographics, diabetic related characteristics and complications. Information on diagnosis of diabetic complications including the location, time and details of the diagnosis was collected. The diagnosis of diabetic complications was checked with patients' medical charts, and confirmed by doctors during the investigation. All subjects were asked to have a HbA1c test free of charge. The blood samples of 5 μl were sent to the assigned center in each city for HbA1c test using the unique procedure with Bio-Rad Variant II. The level of glycemic control was defined as optimal (HbA1c < 6.5%), fair (6.5% ≤ HbA1c ≤ 7.5%), and poor (HbA1c > 7.5%)[18].

This study was approved by the Institutional Review Board of School of Public Health, Fudan University (Approval No: #06-12-0065). Written informed consent was received from all the subjects.

### Complications

Only chronic complications (categorized as cardiovascular conditions, cerebrovascular conditions, nephropathy, ocular lesions, neuropathy, and diabetic foot problems) that developed after the proper diagnosis of T2DM and could be attributed to diabetes were considered in this study. Cardiovascular morbidity included: hypertension, angina, chronic heart failure, myocardial infarction, other related heart diseases, and peripheral vascular disease; the considered cerebrovascular conditions were stroke and transient ischemic attack (TIA); ocular lesions consisted of retinopathy, cataract and blindness; nephropathy included microalbuminuria, macroalbuminuria, renal hypofunction, and renal failure; and diabetic foot problems presented as foot ulcers or amputation (AMP). According to the involved blood vessels, complications were also stratified into macrovascular complications (all cardiovascular, cerebrovascular and foot diseases) and microvascular complications (nephropathies, neuropathy and eye lesions)[19].

### Data management and statistical analysis

All questionnaire data was double-entered and cross-validated using EpiData version 3.1 (EpiData Association, Odense, Denmark). Statistical analyses were performed in SAS version 6.1 (SAS Institute Inc., Cary, USA). Continuous variables were summarized with mean and median, and categorical variables as percentages. Pearson and trend χ^2^-tests were used to explore associations in categorical and ordinal data, respectively. Student's *t-*test was used to compare two means in numerical data. Two-tailed tests were performed with the significance level at 0.05. Generalized linear modeling was employed to estimate the age-adjusted prevalence of complications by time since the first diagnosis of diabetes, and the linear relationship between prevalence of complications and diabetes duration.

## Results

### General characteristics of study subjects

Overall, a total of 1,524 diabetic individuals were eligible in the study, 373 were recruited in Shanghai, 375 in Beijing, 376 in Guangzhou and 400 in Chengdu. Among them were 637 (41.8%) males and 887 (58.2%) females with a mean age of 63.3 years (range: 18 - 88 years). The mean of time span between the diagnosis of T2DM and enrolment in the study was 8.7 years (median: 7.2 years, 25-75th percentile: 3.2-12.5 years).

### Prevalence of chronic complications

Overall, 732 of the 1,524 T2DM subjects (48.0%) had no recognized complications while 792 (52.0%) suffered from at least one diagnosed chronic complication. The categorized prevalence of the chronic conditions is presented in Table 1, along with the prevalence of multiple complications. The prevalence of cardiovascular and cerebrovascular complications, neuropathy, nephropathy, ocular lesions and diabetic foot disease were 30.1%, 6.8%, 17.8%, 10.7%, 14.8% and 0.8%, respectively. The most prominent cardiovascular condition was angina (14.2%), and 5.1% of subjects having cardiovascular condition had two or more conditions. Cerebrovascular complications included 37 cases of stroke, 54 subjects with TIA and 12 individuals suffering both conditions. Of the 162 subjects with nephropathy conditions, 107 had single microalbuminuria, 10 had microalbuminuria and other concurrent nephropathy co-morbidities. A total of 225 subjects suffered from ocular complications, with the most prominent conditions being cataract (9.8%) and retinopathy (6.1%). Foot complications were found among 12 subjects: 10 cases of foot ulcer and 2 individuals who had undergone AMP.

**Table 1:** Prevalence of chronic complications among 1,524 urban Chinese T2DM outpatients, categorized by organ and system

Categories by organ and system	No. of individuals	%
**Cardiovascular conditions**	**459**	**30.1**
**1 cardio-condition**	**381**	**25.0**
Angina	161	10.6
Hypertension	130	8.5
Chronic heart failure	45	3.0
Heart disease	16	1.0
Infarction	12	0.8
Percutaneous transluminal coronary angioplasty	11	0.7
Peripheral vascular disease	6	0.4
**2 cardio-conditions concurrently**	**70**	**4.6**
Angina + other cardio-conditions	47	3.1
Other 2 cardio-conditions concurrently	23	1.5
**3 cardio-conditions concurrently**	**8**	**0.5**
Angina+ other cardio-conditions	8	0.5
**Cerebrovascular conditions**	**103**	**6.8**
**1 cerebro-condition**	**91**	**6.0**
Stroke	37	2.4
Transient ischemic attack(TIA)	54	3.6
**Both stroke and TIA**	**12**	**0.8**
**Neuropathy**	**271**	**17.8**
**Nephropathy conditions**	**162**	**10.7**
**1 nephro-condition**	**152**	**10.0**
Microalbuminuria	107	7.0
Macroalbuminuria	12	0.8
Renal hypofunction	4	0.3
Renal failure	29	1.9
**2 nephro-conditions concurrently**	**10**	**0.7**
Microalbuminuria + other nephro-condition	10	0.7
**Ocular conditions**	**225**	**14.8**
**1 ocular condition**	**205**	**13.5**
Cataract	131	8.6
Retinopathy	73	4.8
Blindness	1	0.1
**2 ocular conditions concurrently**	**20**	**1.3**
Cataract + Retinopathy	19	1.2
Retinopathy + Blindness	1	0.1
**Foot diseases**	**12**	**0.8**
Foot ulcer	10	0.7
Amputation(AMP)	2	0.1
**Single category**	**465**	**30.5**
**2 category concurrently**	**234**	**15.4**
**3 category concurrently**	**74**	**4.9**
**4 plus category concurrently**	**19**	**1.3**
**Total**	**792**	**52.0**

With regard to the prevalence of chronic complications of T2DM across categories (organ or system), 465(30.5%) of the 1,524 subjects had single-category complications while there were 234 (15.4%), 74 (4.9%) and 19 (1.3%) had complications across 2, 3 and 4 plus categories respectively (Table 1). Among the entire study subjects, 33.4% (males: 31.1%; females: 35.1%) had at least one macrovascular complication and 34.7% (males: 28.9%; females: 38.8%) had at least one microvascular complication.

### Geographic and demographic stratification of chronic complications

Table 2 shows the prevalence of chronic T2DM complications, stratified by demographic and geographic variables. The overall prevalence of complications among female subjects was significantly higher than in male subjects (χ^2 ^= 9.75, *p *= 0.002), most notably in neuropathy (χ^2 ^= 12.73, *p *< 0.001) and eye diseases (χ^2 ^= 6.23, *p *= 0.013). The prevalence of complications also varied between subjects from different regions of China (χ^2 ^= 8.763, *p *= 0.033). Higher prevalence of cardio- and cerebrovascular conditions were noted in subjects from Beijing and Guangzhou. Both the overall prevalence of complications (χ^2^_*trend *_= 91.90, *p *< 0.001) and the prevalence of all considered conditions increased with age (*p*-values < 0.05).

**Table 2:** Distribution of chronic complications in different socio-demographic groups among 1,524 urban Chinese T2DM outpatients

Variables	n	Cardiovascular		Cerebrovascular		Neuropathy		Retinopathy		Eye disease		Foot disease		Overall
														
		n (%)		n (%)		n (%)		n (%)		N (%)		n (%)		n (%)
Sex														
Male	637	181 (28.4)		39 (6.1)		87 (13.7)		60 (9.4)		77 (12.1)		6 (0.9)		301 (47.3)
Female	887	278 (31.3)		64 (7.2)		184 (20.7)		102 (11.5)		148 (16.7)		6 (0.7)		491 (55.4)
														
		*χ*^*2 *^= 1.51		*χ*^*2 *^= 0.70		*χ*^*2 *^= 12.73		*χ*^*2 *^= 1.69		*χ*^*2 *^= 6.23		*χ*^*2 *^= 0.33		*χ*^*2 *^= 9.75
		*p *= 0.219		*p *= 0.402		*p *< 0.001		*p *= 0.194		*p *= 0.013		*p *= 0.563		*p *= 0.002

City														
Beijing	375	115 (30.7)		31 (8.3)		57 (15.2)		45 (12.0)		63 (16.8)		2 (0.5)		207 (55.2)
Guangzhou	376	148 (39.4)		31 (8.2)		82 (21.8)		39 (10.4)		42 (11.2)		3 (0.8)		211 (56.1)
Shanghai	373	79 (21.2)		26 (7.0)		58 (15.5)		39 (10.5)		52 (13.9)		2 (0.5)		175 (46.9)
Chengdu	400	117 (29.3)		15 (3.8)		74 (18.5)		39 (9.8)		68 (17.0)		5 (1.3)		199 (49.8)
														
		*χ*^*2 *^= 29.62		*χ*^*2 *^= 8.44		*χ*^*2 *^= 7.29		*χ*^*2 *^= 1.11		*χ*^*2 *^= 6.88		*χ*^*2 *^= 1.71		*χ*^*2 *^= 8.76
		*p *< 0.001		*p *= 0.038		*p *= 0.063		*p *= 0.776		*p *= 0.076		*p *= 0.635		*p *= 0.033

Age (years)														
< 50	147	16 (10.9)		1 (0.7)		8 (5.4)		8 (5.4)		8 (5.4)		-		33 (22.4)
50 ~	391	108 (27.6)		23 (5.9)		36 (9.2)		41 (10.5)		50 (12.8)		1 (0.3)		176 (45.0)
60 ~	552	174 (31.5)		40 (7.2)		105 (19.0)		60 (10.9)		72 (13.0)		5 (0.9)		294 (53.3)
70 ~	434	161 (37.1)		39 (9.0)		122 (28.1)		53 (12.2)		95 (21.9)		6 (1.4)		289 (66.6)
														
		*χ*^*2*^_*trend *_= 32.30		*χ*^*2*^_*trend *_= 11.09		*χ*^*2*^_*trend *_= 65.49		*χ*^*2*^_*trend *_= 3.95		*χ*^*2*^_*trend *_= 25.62		*χ*^*2*^_*trend *_= 4.54		*χ*^*2*^_*trend *_= 91.90
		*p *< 0.001		*p *= 0.001		*p *< 0.001		*p *= 0.047		*p *< 0.001		*p *= 0.033		*p *< 0.001

### Association between chronic complications and T2DM duration

**Figure 1:**
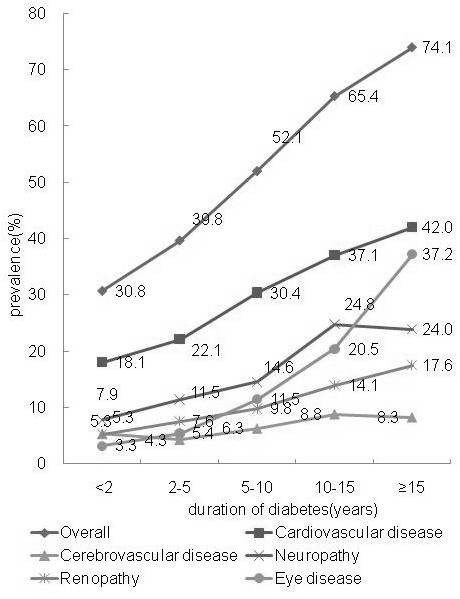
**Age-adjusted prevalence of chronic complications of T2DM in 1524 urban Chinese T2DM outpatients by diabetic duration**.

Figure 1 shows the age-adjusted prevalence of vascular morbidities by time since T2DM had been diagnosed. After adjusting for age, the overall prevalence of complications significantly increased with disease duration (*χ *^2 ^= 106.290, *p *< 0.001). Cardiovascular disease (*χ *^2 ^= 42.411, *p *< 0.001), neuropathy (*χ *^2 ^= 36.226, *p *< 0.001), eye disease (*χ *^2 ^= 107.069, *p *< 0.001) and renopathy (*χ *^2 ^= 21.537, *p *< 0.001) were significantly associated with disease duration. Cerebrovascular morbidity (*χ *^2 ^= 5.103, *p *= 0.024) also showed a significant increase with diabetes duration, but the overall increase in prevalence was low compared to the other conditions investigated here.

### Status of glycemic control in subjects with chronic complications of T2DM

Of the 1511 subjects completing HbA1c tests, the average level of HbA1c for the 784 with chronic complications was 8.2% (ranging from 4.7% to 14.5%), whereas it was 8.0% (ranging from 4.4% to 16.7%) for the 727 without chronic complications (*t *= 2.429, *P *= 0.015). Of subjects with chronic complications, 12.1% meet the optimal targets of HbA1c < 6.5%, while 63.0% had poor glycemic control with their HbA1c > 7.5%. Results for HbA1c of diabetic subjects categorized by chronic complications are shown in Table 3.

**Table 3:** The glycemic control in diabetic patients with and without chronic complications

T2DM chronic complications*	HbA1c			
	
	Mean ± SD(%)	< 6.5%(%)	6.5%-7.5%(%)	> 7.5%(%)
Without chronic conditions(n = 727)	8.0 ± 1.6	111 (15.3)	213(29.3)	403(55.4)
With chronic conditions(n = 784) ^†^	8.2 ± 1.6	95 (12.1)	195(24.9)	494(63.0)
Cardiovascular conditions(n = 457)	8.0 ± 1.5	64(14.0)	121(26.5)	272(59.5)
Neuropathy(n = 268)	8.0 ± 1.6	40(14.9)	66(24.6)	162(60.4)
Cerebrovascular conditions(n = 103)	8.2 ± 1.7	16(15.5)	23(22.3)	64(62.1)
Nephropathy conditions(n = 160)	8.3 ± 1.7	19(11.9)	34(21.3)	107(66.9)
Ocular conditions(n = 221)	8.6 ± 1.7	19(8.6)	43(19.5)	159(71.9)
Foot diseases(n = 12)	8.1 ± 2.0	3(25.0)	3(25.0)	6(50.0)

*: Results of HbA1c tests were not available for five patients without chronic conditions and 8 with complications.

^†^: Patients with multiple category conditions were counted repeatedly under each category.

## Discussion

Considering the significant impact of chronic complications on T2DM-related morbidity and mortality, and the resulting pressure on health care resources[20,21], understanding the epidemiology of T2DM co-morbidities is of great importance. Over the last two decades, there have been only a handful of small-scale studies dealing with the prevalence of chronic complications among diabetic outpatients in China[22-25]. Most of them were conducted in a single hospital or city and had a limited sample size. Only one study carried out in 2002 in 11 cities across China boasted a large sample size of 4,225 T2DM using both in- and outpatients[26]. It should be noted, however, that in this study, data concerning complications was reported by the 200 designated endocrinologists according to their patients' prescriptions. Also, the prevalence of complications among outpatients was not described[26].

The present multi-center study exclusively targeted T2DM outpatients. The 1,524 individuals with T2DM finally included in this study were recruited from the outpatient department (OPD) of 15 general hospitals in four major cities of mainland China. The findings derived from this study can, thus, be expected to a large extent represent the prevalence of T2DM-related chronic morbidity among the respective outpatients in urban mainland China. We found that complications are highly prevalent among urban mainland Chinese T2DM outpatients. Overall, more than half of the individuals included in this study suffered from at least one chronic complication, and almost one-quarter of them were afflicted by 2 or more categorized conditions. Given the estimated 23.8 million diabetic patients across China, as reported by the International Diabetes Federation and that 95% of these cases are of type 2[1], it must be assumed that there are 11-12 million T2DM patients suffering from at least one diabetic co-morbidity across mainland China, and that about 6 million simultaneously suffer from more than one co-morbidity. Thus, chronic complications of T2DM exert a huge burden on China's health care system. The overall prevalence of macrovascular complications (33.4%) noted in the present study is somewhat lower than the corresponding rates reported from Oklahoma, Warsaw, Havana, Berlin and Australia, but is higher than the rates reported in previous studies conducted in China[27-29]. The overall prevalence of microvascular complications found in this study (34.7%) is higher than both the pooled rates in the WHO Multinational Study of Vascular Disease in Diabetes (WHO MSVDD), in Australia and in other studies from China[27-29]. This suggests that the T2DM patients included in our study had an above-average rate of vascular complications. Microvascular conditions in particular markedly increased over time since diabetes had been diagnosed. As a result, the estimated case burden of T2DM-related macro- and microvascular complications across mainland China could be as high as 8 million[1].

This study also found a predominance of cardiovascular conditions among T2DM patients in China over other morbidities. Studies carried out among British outpatients as well as Chinese inpatients also pointed to cardiovascular conditions as the predominant chronic complication of T2DM[30,31]. Therefore, effective measures for the prevention of cardiovascular complications are essential for reducing overall morbidity due to diabetes. Neuropathy, with a prevalence of 17.8%, was somewhat less common in this study than in studies carried out in Canada, the United States and Sweden[32-34]. The prevalence was also about 4% lower than in another study conducted among urban Chinese patients in 2002[26]. The most surprising finding was the low prevalence of foot diseases at 0.8%, much lower than the 8.0% reported for Asian-Americans[35]. However, it should be noted that the criteria employed to establish foot disease differed between the two studies. In the present study, foot disease was established through a doctor's diagnosis, whereas the previously mentioned study relied on self-reported symptoms[35]. In China, even low prevalence translates into substantial absolute disease burden figures due to the high number of diabetic patients. For example, the number of T2DM patients suffering from foot disease (the least prevalent chronic complication according to this study) could be as high as 200,000. This underscores the need for effective programs for screening, preventing and treating diabetic complications.

Both gender and resident location were found to be significantly associated with the prevalence of chronic complications among urban T2DM outpatients in mainland China. Gender, economic differences, variations in lifestyle and unequal health care system performance may each contribute to the observed differences[36]. Variations are substantial in different cities of China. Take Beijing and Shanghai as examples: people in North China such as Beijing are taller, larger than people in south and east China, and also food in north China is saltier and heavier than those in Shanghai[37]. There are also other differences such as housing, climate, lifestyles (smoking, drinking, etc.), coverage of medical insurance, etc. Such variations and their impacts should be further explored in studies on chronic complication of diabetes to allow tailoring interventions to effectively respond to local needs. The higher prevalence of microvascular complications including neuropathy and eye disease among female diabetic patients corresponds to earlier findings reported in the literature[38,39]. Hormones and factors associated with occupational and social inequalities have been invoked as tentative explanations. With regard to the prevention, management and treatment of neuropathy and eye disease, this suggests that special attention should be paid to female diabetic patients.

The prevalence of the investigated chronic diabetic complications was found to increase with age. This is consistent with the results of other studies[30,39,40], and the prevalence of complications was positively associated with the duration of disease, irrespective of the patients' age. Due to the limitations of the cross-sectional study on causation, we cannot infer that duration of diabetes is a risk factor for chronic complications. But the results do point out that much more attention on prevention for diabetic chronic complications should be paid to diabetic patients with longer disease duration. To gain a better control of chronic complications, treatment and management for T2DM complications in urban Chinese populations should primarily target the highly prevalent populations with chronic complications including older diabetic patients and those with a long history of diabetes.

The incidence of chronic complications in T2DM patients was significantly associated with the degree of hyperglycemia, as measured by the plasma glucose or the HbA1c level. According to a cohort study, a 1% reduction in average HbA1c was associated with reductions of 14% for myocardial infarction and 37% for microvascular complications[41]. This study found that 63.4% of the diabetic patients with chronic complications had poor HbA1c control. Although through a cross-sectional study, we could not conclude that poor glycemic control results in chronic complications, it still triggers a warning to the health authority that there is an urgent need for glycemic management, and the chronic complications of T2DM will worsen under current glycemic status.

Since the present study was hospital-based and the subjects were enrolled in the settings of general hospitals in major cities of China, its results only apply for T2DM-related chronic complications in the population frequenting urban mainland Chinese health-care institutions. Therefore the findings are valid for patients managed by hospitals rather than the general diabetic population. Considering that patients who do not frequent the OPD are most likely to be healthier than their peers who were included in this study, the study possibly overestimates the true prevalence of chronic T2DM-related complications among the diagnosed T2DM outpatients in mainland China. One of the limitations of the study is that purposive sampling was applied instead of random sampling. Service coverage, capacity in treating T2DM and participation intention were taken into account for sampling. The selected hospitals were all tertiary or secondary hospitals with specialized department for diabetes, where majority of T2DM patients under treatment visited. However, the purposive sampling could still affect the impacts of the study, especially for generalization. Another limitation of the study is that we do not have the information on patients who reject to participation. To increase the response, our data collectors waited outside the consultation room, and invited patients consecutively for the interview after they completed the consultation with doctors. Although very few patients rejected to participation, bias might be incurred to some extent.

## Conclusions

The present study provides detailed estimates of the prevalence of T2DM-related chronic complications among outpatients in 4 major urban cities across mainland China. Thus, the results are relevant for the prevention, management and treatment of chronic T2DM-related complications in urban mainland China. A high prevalence of chronic complications was found among outpatients with T2DM, with a predominance of cardiovascular and neuropathic conditions. It is worth noting that a high proportion of T2DM outpatients suffered from two or more categorized conditions concurrently. The mean level of HbA1c in diabetic patients with chronic complications was 8.2% and 63.0% of the patients with T2DM-related complications having a poor glycemic control with their HbA1c > 7.5%. The results suggest that policy and strategy aimed at the management of glycemic control and prevention of T2DM complications should be put forward, and that the management of T2DM patients must not be neglected. The increase of complications with age and time since T2DM diagnosis, as well as geographic variation, all point to a need for flexible and adaptive approaches for the prevention and management of T2DM cases in order to allocate medical resources efficiently and according to the true local burden of disease due to T2DM complications.

## Competing interests

The authors declare that they have no competing interests.

## Authors' contributions

ZLL drafted the manuscript and performed the statistical analysis. CWF, WBW, BX designed the study protocol. All authors organized and carried out the original study. And all authors have read and approved the final manuscript.
